# Vaping and e-cigarettes: A public health warning or a health promotion tool?

**DOI:** 10.3126/nje.v9i4.26960

**Published:** 2019-12-31

**Authors:** Edwin van Teijlingen, Preeti Mahato, Padam Simkhada, Cameron van Teijlingen, Mohammad Asim, Brijesh Sathian

**Affiliations:** 1 Bournemouth University, UK; 2 Manmohan Memorial Institute of Health Sciences, Nepal; 3 Nobel College, Nepal; 4 University of Huddersfield, UK; 5 Independent researcher, UK; 6 Trauma Surgery, Surgery, Hamad General Hospital, Doha, Qatar

Over the past decade the use of electronic cigarettes (e-cigarettes) or so-called vaping has become increasingly popular in many parts of the world. The e-cigarette is a battery-operated device which releases vapours of flavoured nicotine instead of tobacco smoke. The e-cigarette is a way to consume nicotine which gives the ‘high’ without the consumption of tar and other cancerous chemicals normally present in conventional tobacco cigarettes. Public Health England reported that smoking e-cigarettes is 95% less harmful than smoking tobacco [1]. Perhaps the e-cigarette is a more pleasant means of nicotine consumption than other nicotine replacement therapies (NRT) such as skin plasters with nicotine or nicotine chewing gum. Particularly, in public health conscious countries like the United Kingdom (UK) and the Netherlands where tobacco is highly taxed, vaping is a cheaper alternative of nicotine consumption. Also in other countries vaping has slowly found its way into the local market. For instance, in the United Arab Emirates (UAE) a recent amendment in the law has legalize the sale of e-cigarettes, whilst local consumers had been buying devices and refills online for some time [2].

## Vaping/e-cigarettes as a public health tool

Earlier the health promoters have considered the use of e-cigarettes to help people quit tobacco smoking. The public health view is that vaping can be an alternate mechanism to relieve the smoker’s craving for nicotine and thus reducing the tar inhalation as few real cigarettes are smoked. To objectively analyse this fact, a Cochrane review brought together several studies which suggested that using e-cigarettes for smoking cessation is more effective than using a placebo, whilst there is one Randomised Controlled Trial (RCT) which reported no significant difference for smoking cessation between use of e-cigarettes versus other substitutes of nicotine for NRT [3].

Relatively recent developments in NRT and more precisely, e-cigarettes have changed our attitudes towards smoking cessation a little. E-cigarettes are becoming more popular among smokers as a way of reducing or cessation of smoking and for minimizing the cost of nicotine intake [1]. This has introduced the notion of harm reduction for smoking cessation. In most countries public health officials have long been arguing along the lines of abstinence from smoking. However, this is different from the public health approach to alcohol use. Whilst, there is evidence that light or ocassional drinking does not seems to have potential harm and may even pose some health benefits, still there is no such evidence regarding cigarette smoking. In addition, heavy smoking is a public health concern which has several adverse effects.

## Risks associated with e-cigarettes

Currently researchers have investigated the risks associated with vaping; as the chemicals used for the flavouring may contain unknown toxic substances which might have long-term side effects. As the potential long-term harm of inhalation of various chemicals through vaping is not fully identified, many public health practioners preferred more traditional methods of smoking cessation (NRT in the form of plasters or chewing gum) over the use of e-cigarettes [4]. It has been suggested that those who fail tobacco smoking cessation by NRT, may switch to e-cigarettes due to expected lesser harm [5].

On the other hand, considering the possible risk of toxic fume generation in e-cigarettes, there is an increasing concern to ban vaping in many countries worldwide; the same way as smoking and use of other tobacco products. Moreover, vaping can also be considered as a so-called gateway to drugs as stated recently by a representative of the National Health Commission in China, who was quoted as saying: “e-cigarettes can easily lure youngsters and turn them into users of traditional tobacco later” in a newspaper article in *China Daily*. Although, e-cigarettes have improved the nicotine delivery, still it’s addictive proterty is not clear so far and thus further research is needed for better understanding [1]. The evidence suggests that although vaping is less harmful than smoking, it is still not risk-free; as it may contain various substances that may adversely impact health and so tobacco smokers switching to e-cigarettes as NRT should stop smoking completely [1,7]. The main challenge for assessment of the health-related effects of e-cigarettes is the marked variety of flavours used in e-cigarette which could have resulted in generation of different aerosol constituents that need to be investigated for harmful effects on public health [8].

## Vaping in WHO South-East Asia Region (WHO-SEAR)

Among the eleven member states in the WHO South-East Asia region; six countries namely North Korea, Nepal, Sri Lanka, Thailand, East Timor and India has banned e-cigarettes. In India, previously e-cigarettes are available in different flavours mostly through the online retail shopping websites [9]. One review published on vaping in India reported a total of 75 companies selling e-cigarettes online [10]. This study also reported the increasing popularity of e-cigarettes among current smokers and especially among adolescents because of its modifiability and similarity with conventional cigarettes. In the earlier publications of 2014 the Ministry of Health and Family Welfare has a firm opinion for prohibiting e-cigarettes in India [11] and from the public health perspective there are indications that vaping may become illegal in India [12,13]. Hence, following the footsteps of other Southeast Asian countries, recently India has announced the ban on e-cigarettes in the entire country in September 2019 [14].

However, other WHO-SEAR such as Bangladesh, Bhutan, Indonesia, Maldives, Myanmar have not imposed a ban on production, import and sale of e-cigarettes, till date. Owing to the health risks and teen addiction of vaping, Bangladesh is actively planning to ban e-cigarette under the tobacco control policy [15]. In Bhutan, use of e-cigarette is still legalized despite regulations for tobacco and tobacco products use which can be imported; but subjected to import duties and display health warnings [16]. Countries like Indonesia which is the world’s second largest tobacco market is also planning to impose complete ban on e-cigarettes in anticipation of the growing health concerns associated with vaping. In Myanmar, despite the promotion of anti-smoking campaigns and control on the sale of vapes and e-cigarettes, regulations on complete ban of vaping will still need some more time. E-cigarettes are regulated similar to other tobacco products in Maldives available with health warnings and such electronic nicotine delivery systems (ENDS) are prohibited in places where smoking is restricted [17].

Worldwide, e-cigarettes are becoming increasingly popular among smokers as a substitute for reducing or cessation of smoking. It relieves from the desire to tobacco smoking and help in cutting down the number of cigarettes smoked which is seen as a key advantage of the e-cigarette use, particularly among heavy smokers. The UK’s positive policy approach to e-cigarettes creates an important environment for researching their impact [1]. Currently, there is limited data available from the WHO-SEAR, and so further research is needed to address the following issues:

- What is the long-term safety of e-cigarettes, and are they as safe as other NRT products?
- Are e-cigarettes a gate-way into tobacco use?
- How can we educate potential and actual users effectively about potential risks of using e-cigarettes?
- Is using e-cigarette a cost effective way of smoking cessation, and is it as effective as other NRTs?
- What is the impact of health promotion interventions to change perceptions of the relative harmfulness of e-cigarettes and NRT compared with cigarettes?
- What is the role of vape shops in helping smokers to quit tobacco?
- What is the role of price, promotion and availability in the use of e-cigarettes by smokers?
- What are the implications of e-cigarette ban in WHO-SEAR countries on the effectiveness of tobacco cessation?

In conclusion, the potential benefits and harm of using e-cigarette or vaping should be considered before using it as a potential public health tool for tobacco cessation. Considering the negative effects of vape use on heart and lungs, many WHO-SEAR has imposed a strict ban on e-cigarettes and others are also planning to bring regulations in the near future. Further investigations for better understanding of this important public health issue is urgently needed.
